# Development of a microplate coagulation assay for Factor V in human plasma

**DOI:** 10.1186/1477-9560-9-11

**Published:** 2011-06-28

**Authors:** Derek Tilley, Irina Levit, John A Samis

**Affiliations:** 1 Applied Bioscience, Faculty of Science, University of Ontario Institute of Technology, Oshawa, ON. L1H7K4. Canada; 2 Faculty of Health Sciences, University of Ontario Institute of Technology, Oshawa, ON. L1H7K4. Canada

## Abstract

**Background:**

Factor V (FV) in its activated form, FVa, is a critical regulator of thrombin generation during fibrin clot formation. There is a need of a simple, fast, and inexpensive microplate-based coagulation assay to measure the functional activity of FV in human plasma. The objective of this study was to develop a microplate-based assay that measures FV coagulation activity during clot formation in human plasma, which is currently not available.

**Methods:**

The FV assay requires a kinetic microplate reader to measure the change in absorbance at 405nm during fibrin formation in human plasma. The FV assay accurately measures the time, initial rate, and extent of fibrin clot formation in human plasma.

**Results:**

The FV microplate assay is simple, fast, economical, sensitive to approx 24-80pM, and multiple samples may be analyzed simultaneously. All the required materials are commercially available. Standard curves of time or initial rate of fibrin clot formation vs FV activity in the 1-stage assay (Without activation by thrombin) may be used to measure FV activity in samples of human plasma. The assay was used to demonstrate that in nine patients with disseminated intravascular coagulation (DIC), the FV 1-stage, 2-stage (With activation by thrombin), and total (2-stage activity - 1-stage activity) activities were decreased, on average, by approximately 54%, 44%, and 42%, respectively, from prolonged clot times when compared to normal pooled human reference plasma (NHP). The results indicate that the FV in the DIC patient plasmas supported both a delayed and slower rate of fibrin clot formation compared with NHP; however, the extent of fibrin clot formation in the DIC patients remained largely unchanged from that observed with NHP.

**Conclusions:**

The FV microplate assay may be easily adapted to measure the activity of any coagulation factor using the appropriate factor-deficient plasma and clot initiating reagent. The microplate assay will find use in both research and clinical laboratories to provide measurement of the functional coagulation activity of FV in human plasma.

## Background

The use of microplate-based assays for quantification of enzyme activity is now common practice in many laboratories for enzyme linked immunosorbant assays [1] and colorimetric enzyme assays using chromogenic substrates [2]. The two main advantages for microplate-based assays are use of small sample volumes and that multiple samples may be analyzed simultaneously. Several microplate assays have been previously reported for measurement of clot lysis [3], platelet aggregation [4], the activity of Factor (F) VII, VIII, IX and IXa, X, XI, XII, and activated protein C (APC) [5,6]. General coagulation and fibrinolysis microplate assays have also been described [7]. Finally, although manual [8,9] and automated [10] FV coagulation assays have been described, no microplate-based coagulation assay has been previously reported to measure FV activity in human plasma.

FV circulates in humans at approx 12-40nM as an inactive procofactor of Mr 330kDa [11]. In response to injury, activation of FV to the activated cofactor, FVa, occurs initially by thrombin [12]. FVa is a major regulatory component of coagulation where it accelerates thrombin generation and consequent fibrin clot formation as part of the *prothrombinase *enzyme complex [13]. In the presence of thrombomodulin, thrombin activates protein C to activated C (APC) which inactivates FV and FVa and downregulates coagulation as part of the normal anticoagulation response to injury [14]. The FV Leiden mutation (Arg 506 to Gln) results in FVa becoming resistant to APC inactivation and results in a hypercoagulable state in affected individuals [15]. The FV Leiden mutation is currently the most common genetic risk factor for venous thrombosis in humans [16].

Although methods for determining the FV 1-stage activity (Without activation by thrombin) in human plasma using manual tilt-tube assays have been described [8,9], the results may be prone to operator bias. Further, these FV assays are time consuming because only one sample may be assayed at a time. Since FV circulates in plasma as an inactive procofactor [11], it must be 'intentionally' activated with added thrombin to generate FVa for accurate and complete measurement of the FV 2-stage activity (With activation by thrombin). The total FV activity may then be calculated using the measurements from FV 1- and 2-stage assays (Total FV activity = 2-stage activity - 1-stage activity). Methods for determining the FV 2-stage activity in plasma using tilt-tube [8,9] and automated [10] formats have been described.

The equipment required to perform automated coagulation assays is expensive. Although these analyzers are generally used in clinical laboratories to measure clot times in patient plasmas, the rate and extent of clot formation are not routinely assessed. The FV microplate assay described here provides measurement of the time, initial rate, and extent of clot formation in human plasma in a fast and convenient manner. It may be easily adapted to measure the activity of any coagulation factor in the extrinsic, intrinsic, or common pathway using the appropriate factor-deficient plasma and clot initiating reagent. This new microplate-based coagulation assay will be useful in both research and clinical laboratories to increase our understanding of the biochemistry and functional activity of FV.

## Materials and methods

### Materials

Normal pooled human reference plasma (NHP) was obtained from Precision Biologicals (Dartmouth, NS, Canada) and was stored at -80°C until use. Fresh frozen human plasma was obtained from Lakeridge Healthcare (Oshawa, ON, Canada) and stored at -80°C until use. FV-deficient plasma was made from fresh frozen human plasma according to Bloom *et al*. [9], dialyzed exhaustively against 20mM HEPES, 0.15M NaCl, pH 7.4 (HBS), and stored at -80°C until use. The FV-deficient plasma contained less than 0.1% of the active FV present in the original fresh frozen human plasma. Thromboplastin reagent from Trinity Biotech (Wicklow, Ireland) was reconstituted with and dialyzed exhaustively against HBS at 4°C, and stored at -80°C until use. Dialysis was required to remove any calcium in this reagent in order to permit precisely timing the reaction from the time of addition of calcium chloride to initiate clotting (See below). Purified thrombin was prepared according to Bajzar *et al*. [17]. The nine patients studied were diagnosed with disseminated intravascular coagulation (DIC) at some point during their stay in the Intensive Care Unit of The Royal Liverpool University Hospital (Liverpool, UK) according to The International Society on Thrombosis and Haemostasis Scientific and Standardization Committee algorithm [18]. Patients were treated according to standard procedures and none received heparin or anticoagulant factor concentrates. Patient blood samples were drawn into 0.1 volume of 3.8% sodium citrate according to The Liverpool Research Ethics Committee and the plasma fractions were stored at -80°C until use. To determine the normal range of FV activity in the FV 1-stage microplate assay, approximately 4.5 ml of blood was drawn from 15 healthy subjects (Age 18-22 years) from the Medical Laboratory Science Program at The University of Ontario Institute of Technology into 0.5 ml of 3.8% sodium citrate and the plasma fractions were stored at -80°C until use. The research complied with all relevant laws, guidelines, and policies. Thawed/refrozen aliquots of NHP, FV-deficient plasma, thromboplastin, and patient plasma were not used for study.

### Factor V Coagulation Assays

The FV microplate assay was performed in the wells of immunomodule strips (Nunc, Roskilde, Denmark) using a multichannel pipette to make simultaneous additions of FV-deficient plasma (50 μl), followed by normal or patient plasma diluted in HBS (50 μl), and finally thromboplastin (50 μl). The samples were incubated in a Spectra Max 190 microplate reader (Molecular Devices, Sunnyvale, CA, USA) at 25°C for 1 minute with shaking during the 15-25 seconds of the 1 minute incubation. Calcium chloride (50 μl of 25mM) was then added to initiate clotting. The samples were then shaken for 10 seconds and the absorbance at 405nm was read every 5 seconds for 6 minutes. The 10 seconds required for shaking prior to absorbance readings were not included in the calculated clot times. The clot times were calculated as the time to reach the midpoint between the minimum and maximum absorbance at 405nm after calcium chloride addition. The initial rate of clot formation was calculated as the rate of increase in absorbance at 405nm (mUnits/minute) using the first 5 time points of clot formation in the linear portion of the absorbance vs time curve. The extent of clot formation was calculated as the difference between the maximal and minimal absorbance in Units at 405nm achieved during the clot formation event.

The FV 1-stage activity of a plasma sample diluted 40-fold in HBS was determined by comparison to the FV 1-stage microplate assay standard curves of times or initial rates of clot formation vs FV activity measured upon serial dilution of NHP (0- to 1024-fold) in HBS. One unit of FV activity was defined as the activity in 1 ml of NHP prior to activation by thrombin (See below). Up to 12 samples were analyzed simultaneously using SOFTmax PRO 4.3 LS software (Molecular Devices, Sunnyvale, CA, USA). The above microplate assay measures the FV 1-stage activity (Without activation by thrombin) of a plasma sample. Since FV is activated with thrombin to the active cofactor, FVa [12], a second assay after addition and incubation with thrombin was critical to accurately measure the FV 2-stage (With activation by thrombin) cofactor activity of a plasma sample. For the FV 2-stage assay, purified thrombin (1 Unit/ml; 10nM final concentration) was added to the plasma sample diluted 100-fold in HBS and incubated for 1 minute at 37°C. The plasma sample was diluted a further 5-fold in HBS and re-assayed at a 500-fold dilution as described above. The FV 2-stage activity was interpolated from the FV 1-stage assay standard curve of time of clot formation vs FV activity (See above). The total FV activity was calculated from the measured values from the FV 2-stage and 1-stage assays and was defined as the 2-stage activity - 1-stage activity.

The results shown for the times and initial rates of clot formation in the FV 1-stage assay standard curves are representative of experiments conducted on greater than thirty different occasions. The intra- and inter-FV assay variability of the time, extent, and initial rate of clot formation were calculated from six samples of NHP diluted 40-fold in HBS on eight different days.

## Results

**Figure 1:**
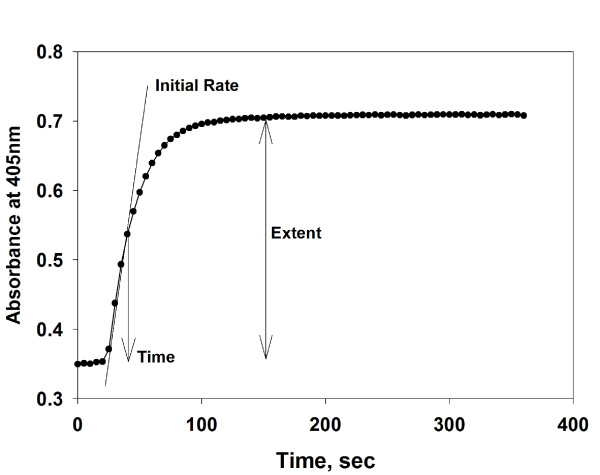
**Monitoring clot formation in normal pooled human reference plasma with the kinetic microplate FV 1-stage coagulation assay**. Fibrin clot formation in NHP was monitored at 405nm over time in a microplate reader. The profile represents the actual microplate reader output of a 6 minute reaction of 32-fold diluted NHP, FV-deficient plasma, thromboplastin, and calcium chloride in a single microplate well. The vertical axis denotes the change in absorbance at 405nm that occurred as a result of clot formation in plasma. The time of fibrin formation was defined as the time to reach the half maximal increase in absorbance; approximately the midpoint of curve (36.40 seconds). The initial rate of clot formation was defined as the rate of change of absorbance at 405nm over the first 5 time points of the linear increase of absorbance portion of the curve (611.88 mUnits/min). The extent of clot formation was defined as the difference between the maximum and minimum absorbance at 405nm (0.35 Units).

A typical depiction of clot formation in the FV coagulation assay over time generated by the microplate reader is shown in Figure 1. Visual inspection of the wells after the assay was performed confirmed that clot formation took place. All reactions reached approximately the same extent of clot formation with a typical change in absorbance at 405nm of 0.35 - 0.45 Units between the starting absorbance before and the maximal absorbance after thromboplastin and calcium chloride addition.

**Figure 2:**
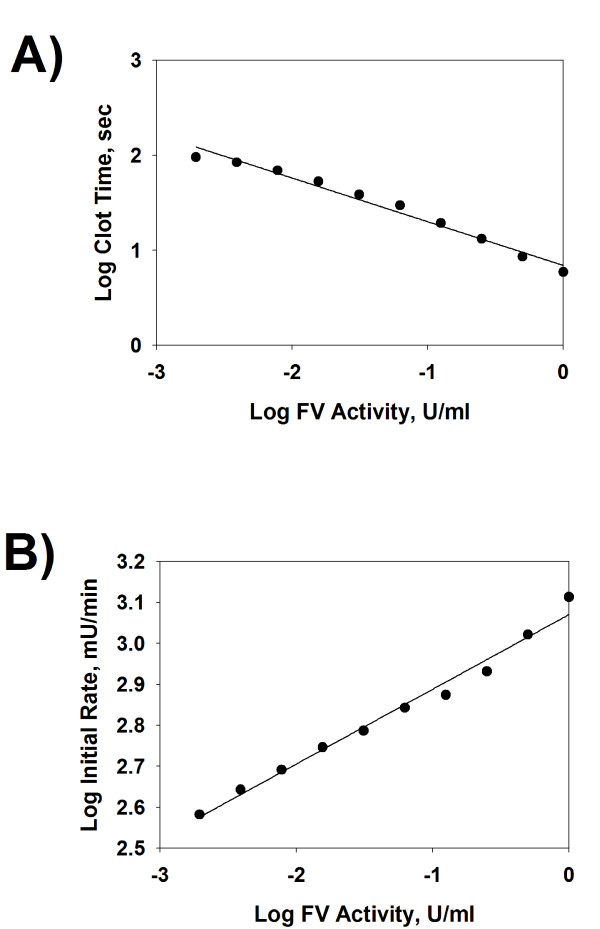
**Standard curves of time and initial rate of clot formation vs Factor V activity in normal pooled human reference plasma using the FV 1-stage microplate assay**. NHP was serially diluted (0- to 1024-fold in HBS) and assayed with the FV 1-stage microplate assay as described in the text. Log-Log plots of the times and initial rates of clot formation vs FV activity in the FV 1-stage microplate assay are shown after linear regression modelling of the results in panels A and B, respectively.

In order to measure the FV activity in a plasma sample, standard curves of time and initial rate of clot formation vs FV 1-stage activity were generated upon serial dilution of NHP. Fitting the Log-Log plot of clot time vs FV 1-stage activity demonstrated a strong linear relationship between these variables after regression analysis (Figure 2A; R^2 ^= 0.980). Fitting of the Log-Log plot of the initial rate of clot formation vs FV 1-stage activity also demonstrated a strong linear relationship between these variables after regression analysis (Figure 2B; R^2 ^= 0.983). The relationship of both clot time and initial rate of clot formation vs FV 1-stage activity remained linear in NHP diluted up to approx 1024-fold. Given that the FV concentration in NHP is approx 12-40nM [11], the results indicate that the assay is sensitive to approximately 24-80pM FV in NHP. Our results indicate that the normal range of FV activity in the FV 1-stage activity assay in 15 healthy controls assayed with the FV microplate assay was approximately (Mean ± Standard deviation; Range): 0.96 ± 0.14 U/ml; 0.68-1.11 U/ml. This is similar to the FV activity and range (0.66-1.14 U/ml) in healthy controls reported by Cutler *et al*. for the FV 1-stage activity measured with an automated analyzer [19].

Under the conditions of the assay using 40-fold diluted NHP, the intra-assay variability of the time, extent, and initial rate of clot formation in the FV 1-stage assay among 6 wells on 8 different days was 3.4%, 4.4%, and 3.1%, respectively. The inter-assay variability of the time, extent, and initial rate of clot formation in the FV 1-stage assay among 6 wells of 8 different experiments on 8 different days was 7.1%, 7.8%, and 9.2%, respectively. Thus, the intra- and inter-assay variability of these three variables in the FV 1-stage assay was at a low and acceptable level.

The standard curve of clot time vs FV 1-stage activity (Figure 2A) was used to measure the FV activity in nine DIC patient plasmas which were (FV 2-stage activity) or were not (FV 1-stage activity) intentionally activated with thrombin and the results are shown in Table 1. All nine DIC patient plasmas exhibited FV 1-stage activities and initial rates of clot formation that were decreased on average, by approx 54% and 18%, respectively, from NHP. The extents of clot formation in the FV 1-stage assay in the DIC patients were not largely different from NHP, and increased on average, by approx 13% from NHP.

**Table 1:** FV Activity in NHP and in 9 Patients That Developed Disseminated Intravascular Coagulation (DIC)

Sample	1-stage assay Activity (Units/ml)	1-stage assay Extent (Units)	1-stage assay Initial Rate (mUnits/min)	2-stage assay Activity (Units/ml)	2-stage assay Extent (Units)	2-stage assay Initial Rate (mUnits/min)	Total activity (Units/ml)
**NHP**	1.02	0.363	744.96	7.93	0.433	375.84	6.91
**Patient 1**	0.36	0.404	583.80	3.84	0.432	249.96	3.48
**Patient 2**	0.67	0.416	704.04	5.28	0.469	323.16	4.61
**Patient 3**	0.31	0.435	562.08	3.92	0.453	294.96	3.61
**Patient 4**	0.39	0.435	617.04	4.14	0.462	372.60	3.75
**Patient 5**	0.73	0.401	641.40	5.91	0.433	354.24	5.18
**Patient 6**	0.45	0.403	600.72	4.16	0.445	393.96	3.71
**Patient 7**	0.49	0.395	575.40	4.89	0.449	357.48	4.40
**Patient 8**	0.19	0.448	489.00	2.89	0.450	330.48	2.70
**Patient 9**	0.64	0.423	699.48	5.11	0.455	417.24	4.47

The FV 1-stage, 2-stage, and total activity in normal pooled human reference plasma (NHP) and the 9 DIC patient plasma samples were determined from the FV 1-stage microplate assay standard curve of time of clot formation vs FV activity (Figure 2A) and are given in Units/ml. The initial rates (Initial rate of increase in A405nm over first five time points in mUnits/min) and extents (Maximum A405nm - Minimum A405nm) of clot formation in the FV 1- and 2-stage microplate assay are also shown.

Activation of NHP with thrombin generated an approximate 8-fold increase in FV 2-stage activity above the FV 1-stage activity (Table 1). This indicates that the FV in NHP was mainly present in its unactivated form and is consistent with the results reported by Nesheim *et al*. using a manual tilt-tube FV assay [8]. The FV 2-stage and total activity were also decreased in the DIC patients on average, by approx 44% and 42%, respectively, from NHP. The initial rates and extents of clot formation in the FV 2-stage assay in the DIC patients were not largely different from NHP, and varied on average, by approx 9% and 4%, respectively, from that observed with NHP.

## Discussion

The kinetic microplate-based assay outlined here describes the development of a new, fast, economical, and simple technique for measurement of FV coagulation activity in samples of human plasma which has or has not been intentionally activated by thrombin. The assay requires a kinetic microplate reader and all the required reagents and materials are commercially available. The assay measures the increase in turbidity of plasma during clot formation at 405nm using a kinetic plate reader. The assay also has the added advantage of using small sample volumes (50 μl) as well as being able to analyze multiple samples simultaneously (up to 12). These assay characteristics are especially important and advantageous when expensive reagents are required such as factor-deficient plasmas; when only small sample volumes are available such as patient plasmas or when analyzing a large number of samples during purification of FV from plasma.

The results indicate that the FV in the DIC patient plasmas was functionally less active than in NHP because of prolonged clot times and decreased initial rates of clot formation in the FV 1-stage assay. However, the initial rates of clot formation in the FV 2-stage assay in the DIC patients were not largely different from NHP. The extents of clot formation in the DIC patient plasmas were also not largely different from NHP in the FV 1-stage and 2-stage assays. Since the extent of clot formation in the DIC patient plasmas was the same as observed with NHP, this was most likely determined by the fibrinogen level in the FV-depleted plasma and unrelated to characteristics of the patient plasmas.

These results indicate that the FV in the DIC patient plasmas supported both a delayed and slower rate of fibrin clot formation compared with NHP; however, the extent of fibrin clot formation in the DIC patients remained largely unchanged from that observed with NHP. Compared with NHP, the FV in the DIC patient plasmas was also on average, 44% less activatable with thrombin in the FV 2-stage assay. Finally, the FV total activity in the DIC patients decreased by approx 42% on average, from that observed in NHP. The decreased FV 1-stage, 2-stage, and total activities may have been due to increased FV consumption [20] and/or inactivation [21] which occurred during the pathogenesis of this acquired blood disorder in the patients studied. Our preliminary results indicate that the FV antigen levels in the DIC patient plasmas studied were not significantly different from NHP [11], which indicates a qualitative disorder of FV occurred in this condition.

The results of the microplate assay indicate that measurement of FV coagulation activity in human plasma samples may be obtained from the time and initial rate measurements of clot formation from the FV 1-stage assay and the time of clot formation and total activity measurement from the FV 2-stage assay. It was however not possible to obtain a quantitative measurement of FV coagulation activity in a plasma sample based on the extent of clot formation in the FV 1- and 2-stage assays or the initial rate of clot formation in the FV 2-stage assay. Since all reactions reached approximately the same extent of clot formation at 405nm of 0.35 - 0.45 Units between the starting absorbance before and the maximal absorbance after thromboplastin and calcium chloride addition, measurement of the extent of clot formation does not strictly provide a quantitative measurement of FV coagulant activity in any given plasma sample.

The new microplate assay will be useful in both clinical and research laboratories for measurement of FV activity in human plasma samples. The microplate assay may be used to measure the time, initial rate, and extent of clot formation; parameters not routinely monitored simultaneously in most automated coagulation analyzers. In its entirety, this quantitative information provides more of a complete 'picture' of the involvement of FV during the clot formation event in human plasma than has been previously reported [5,7-10,21]. This information, in turn, may be especially important in both research and clinical settings when assessing significant changes in FV activity in plasma samples or using purified FV or FVa. Other applications of the FV assay include its use to detect and measure compounds and substances that may activate or inactivate FV or FVa, measure the FV activity in patients at risk for venous thrombosis as a result of the FV Leiden mutation [15,16], and to measure the FV activity during its purification from either human or animal plasma.

## Conclusion

The FV microplate assay format may be easily adapted to measure the activity of any coagulation factor in the extrinsic, intrinsic, or common pathway using the appropriate factor-deficient plasma and clot initiating reagent. The clinical and research use of this new microplate-based coagulation assay will provide further information and understanding of FV biochemistry and functional activity in the future.

## Declaration of Competing interests

The authors declare that they have no competing interests.

## Authors' contributions

JAS supervised the study design, interpretation of results, manuscript preparation and wrote the manuscript. DT acquired and analyzed the data and helped write the manuscript. IL assisted DT in acquiring and analyzing some of the data and helped write the manuscript. All authors read and approved the final manuscript.
